# Altered expression of synaptic proteins and adhesion molecules in the hippocampus and cortex following the onset of diabetes in nonobese diabetic mice

**DOI:** 10.14814/phy2.15673

**Published:** 2023-04-20

**Authors:** Takumi Yokokawa, Kohei Kido, Koji Sato, Tatsuya Hayashi, Satoshi Fujita

**Affiliations:** ^1^ Laboratory of Sports and Exercise Medicine, Graduate School of Human and Environmental Studies Kyoto University Kyoto Japan; ^2^ Research Organization of Science and Technology Ritsumeikan University Kusatsu Shiga Japan; ^3^ Division of Food Science and Biotechnology, Graduate School of Agriculture Kyoto University Kyoto Japan; ^4^ Faculty of Sport and Health Science Ritsumeikan University Kusatsu Shiga Japan; ^5^ Faculty of Sports and Health Science Fukuoka University Fukuoka Japan; ^6^ Graduate School of Human Development and Environment Kobe University Kobe Japan

**Keywords:** insulin, synapse, synaptic adhesion molecules, type 1 diabetes

## Abstract

Mounting evidence links Type 1 diabetes (T1D) with cognitive dysfunction, psychiatric disorders, and synaptic alterations; however, the underlying mechanism remains unclear. Numerous synaptic proteins and synaptic adhesion molecules (SAMs) that orchestrate synaptic formation, restructuring, and elimination are essential for proper brain function. Currently, it is unclear whether the pathogenesis of T1D is related to the expression of synaptic proteins and SAMs. Here, we investigated whether T1D mice exhibited altered synaptic protein and SAM expression in the hippocampus and cortex. We discovered that T1D mice exhibited partially decreased levels of excitatory and inhibitory synapse proteins and SAMs, such as neurexins, neuroligins, and synaptic cell adhesion molecules. We also found that compared to control mice, T1D mice showed a marginal decrease in body weight and a significant increase in plasma glycoalbumin levels (a hyperglycemia marker). These results provide novel molecular‐level insights into synaptic dysfunction in mice with T1D.

## Graphical abstract

We examined the effect of Type 1 diabetes (T1D) on the synaptic proteins and adhesion molecules. T1D mice exhibit decreased expression of some synapse proteins. T1D mice have reduced expression of synaptic adhesion molecules.

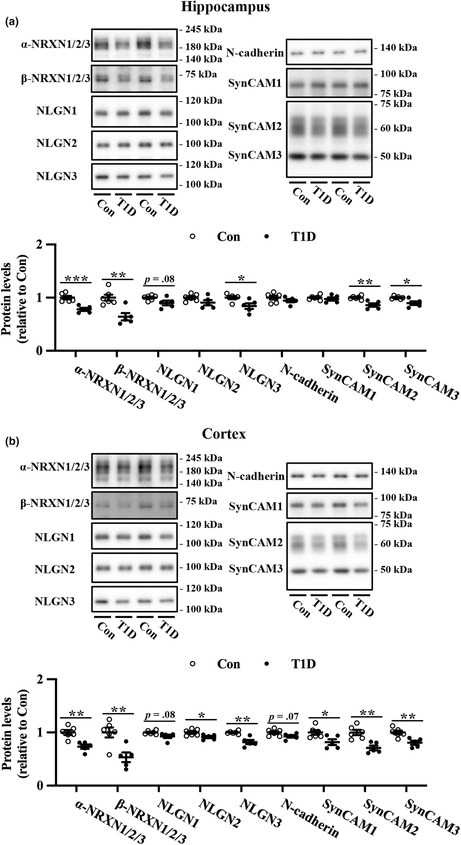

## 1 INTRODUCTION

Mounting clinical evidence links Type 1 diabetes (T1D) with abnormal brain function. Patients with T1D exhibit cognitive deficits and depression (Gilsanz et al., 2017; Hansen et al., 2017; Ryan, 1988; Silverstein et al., 2015; Trief et al., 2014). Similarly, mice with T1D demonstrate learning impairments and depression‐like behaviors (Biessels et al., 1996; Flood et al., 1990; Hilakivi‐Clarke, 1991). An electrophysiological study showed impaired synaptic plasticity in the hippocampus of T1D model mice (Valastro et al., 2002). However, the molecular mechanisms underlying the impaired brain function and synaptic plasticity in individuals with T1D remain unclear.

Synaptic molecules are required to maintain integral synaptic plasticity and brain function. Mice with a knockout of specific excitatory or inhibitory synapse‐localized proteins exhibit impaired memory function and enhanced anxiety (Crestani et al., 1999; Nakazawa et al., 2002; Sakimura et al., 1995). Synaptic adhesion molecules (SAMs) orchestrate synapse formation, restructuring, and elimination in a synapse type‐specific manner (reviewed in Südhof, 2018). Knockout of SAMs, such as neuroligins (NLGNs) (Blundell et al., 2010; Liang et al., 2015), neurexins (NRXNs) (Anderson et al., 2015; Dachtler et al., 2014; Etherton et al., 2009; Grayton et al., 2013), synaptic cell adhesion molecules (SynCAMs) (Park et al., 2016; Robbins et al., 2010), and neural cadherin (N‐cadherin) (Bozdagi et al., 2010; Nikitczuk et al., 2014), elicits altered synaptic function, neuropsychiatric abnormalities, and impaired memory function.

Peripheral insulin extensively regulates the gene expression of synaptic molecules, including excitatory or inhibitory synapse‐localized proteins (Cai et al., 2021). Therefore, hypoinsulinemia and T1D may elicit abnormal expression of several synaptic molecules, underlying impaired synaptic plasticity and brain function in these contexts. Streptozotocin‐treated rodents, a pharmacologically induced T1D model, exhibit lower expression of synaptophysin, a general synaptic marker, in the hippocampus (Hou et al., 2012). Among the *N*‐methyl‐d‐aspartate (NMDA) glutamate receptor subunits, which are modulators of synaptic plasticity and learning, these rodent models also exhibit decreased protein GluN2B levels (but not GluN1 or GluN2A) in hippocampal postsynaptic densities (Gardoni et al., 2002; Luca et al., 1999). In contrast, in a nonobese diabetic (NOD) mice, a spontaneous T1D model, exhibit an increased GluN2A expression (but not GluN1) in the synaptosome of the hippocampus was noted (Valastro et al., 2002); thus, the effect of T1D on glutamate receptors remains controversial. STZ treatment induces direct damage to the liver and kidney (Kraynak et al., 1995; Kume et al., 2004; Palm et al., 2004), making it difficult to suggest an association between hypoinsulinemia and synaptic molecules. Therefore, further comprehensive studies using NOD mice are required.

Based on the evidence above, we hypothesized that T1D mice have altered expression of synaptic proteins and SAMs in the hippocampus and cortex. To test this hypothesis, we compared the synaptic protein and SAM levels in the hippocampus and cortex of mice with T1D and nondiabetic control mice.

## 2 METHODS

### 2.1 Animals

All animal care procedures were approved by the Committee on Animal Care at Ritsumeikan University. Female 6‐week‐old NOD/ShiJcl mice, which spontaneously develop a T1D‐like phenotype (Makino et al., 1980), were purchased from Japan SLC. Female mice were used to study the onset of diabetes symptoms because they exhibit a higher incidence of diabetes than male mice (Amrani et al., 1998; Makino et al., 1980). Mice were housed under controlled conditions with a 12‐h light–dark cycle and provided food and water ad libitum. Non‐fasting blood glucose levels were monitored using a glucometer (Ascensia Breeze 2, Bayer Healthcare) every morning between 10 and 36 weeks of age to diagnose diabetes. Mice were considered to have T1D when the non‐fasting glucose level was >250 mg/dL for two consecutive days. In contrast, normoglycemic mice were used as nondiabetic controls. After diabetes onset, diabetic mice were maintained on daily insulin (0.6 U, Lantus) to avoid death. At least 4 weeks after onset, overnight‐fasted mice (37 weeks old) were euthanized with isoflurane. Subsequently, the hippocampus and cortex were rapidly excised, frozen in liquid nitrogen, and stored at −80°C until analysis.

### 2.2 Blood analysis

Plasma samples were collected transcardially using a heparin‐treated syringe after overnight fasting and centrifuged at 1000 *×* ***g*** at 4°C for 10 min. Plasma glycoalbumin levels were determined by Oriental Yeast Co., Ltd.

### 2.3 Primary antibodies

The following antibodies were used: Synaptophysin (SYP; 1:5000, Cell Signaling Technology, 5461); Synapsin‐1 (SYN1; 1:10000, Cell Signaling Technology, 5297); synaptoporin (SNPR; 1:1000, Santa Cruz Biotechnology, sc‐376761); vesicular glutamate transport protein 1 (VGLUT1; 1:5000, NeuroMab, 75‐066); VGLUT2 (1:5000, Millipore, MAB5504); GluA1 (1:5000, NeuroMab, 75‐327); GluA2 (1:5000, NeuroMab, 75‐002); GluA3 (1:5000, Abcam, ab40845); GluN1 (1:5000, Abcam, ab109182); GluN2A (1:5000, Abcam, ab133265); GluN2B (1:10000, Abcam, ab183942); postsynaptic density protein 95 (PSD‐95; 1:5000, NeuroMab, 75‐028); Homer1 (1:5000, Abcam, ab184955); vesicular GABA transporter (VGAT; 1:5000, Synaptic Systems, 131011); glutamate decarboxylase 2 (GAD2; 1:5000, Cell Signaling Technology, 5843); gephyrin (1:5000, Abcam, ab177154); glycine receptor (GlyR; 1:5000, Synaptic Systems, 146011); GABA A receptor subunit α1 (GABAARα1; 1:5000, NeuroMab, 75‐136); GABAARβ1 (1:5000, NeuroMab, 75‐137); GABAARβ2/3 (1:5000, Merck, 05‐474); GABAARγ2 (1:5000, Synaptic Systems, 224‐003); NRXN1/2/3 (1:5000, Synaptic Systems, 175003); NLGN1 (1:3000, NeuroMab, 75‐160); NLGN2 (1:10000, Synaptic Systems, 129511); NLGN3 (1:3000, NeuroMab, 75‐158); N‐cadherin (1:1000, BD Biosciences, 610920); and SynCAM1/2/3 (1:5000, Synaptic Systems, 243003).

### 2.4 Western blotting

Western blotting was performed as previously described (Yokokawa et al., 2018). Briefly, the whole hippocampus and cortex were lysed in ice‐cold radioimmunoprecipitation buffer (50 mM Tris–HCl [pH 7.4], 150 mM NaCl, 1% Nonidet P‐40, 1% sodium deoxycholate, 1 mM ethylenediaminetetraacetic acid, 1 mM ethylene glycol tetraacetic acid, and 0.1% sodium dodecyl sulfate [SDS]) supplemented with a protease and phosphatase inhibitor cocktail. Subsequently, lysates were centrifuged at 14,000 *×* ***g*** for 20 min at 4°C. The protein concentration of the supernatants was determined using a Protein Assay Bicinchoninate Kit (Nacalai Tesque). Equal volumes of lysates (0.2–25 μg) were subjected to SDS polyacrylamide gel electrophoresis (7%–13%). The separated proteins were transferred to polyvinylidene difluoride membranes (Millipore). The membranes were blocked with 5% non‐fat dry milk (NFDM) in Tris‐buffered saline‐0.01% Tween 20 (TBST) for 30 min at 25°C and subsequently washed with TBST. Primary antibodies were diluted in 5% bovine serum albumin/TBST and incubated overnight at 4°C. The membranes were subsequently washed and incubated with appropriate secondary antibodies (1:3000, Cell Signaling Technology, 7074 or 7076) in 1% NFDM/TBST for 1 h at 25°C. After the final washes, digital images were obtained using Chemi‐Lumi One L or Ultra (Nacalai Tesque) and LuminoGraph III Light (ATTO). Western blotting was performed in duplicate, and the average values were used for quantification to minimize technical errors. The signal intensities of the target proteins on the immunoblots were normalized to Coomassie brilliant blue staining. To validate the quantitative capability of immunoblots, subjected sample volume (0.2–2.5 μg), antibody concentration (1:500–1:20000), and antibody dilution buffer (BSA or NFDM) were appropriately determined using pilot studies. Since some antibodies have not been validated by knockout or blocking peptide, to evaluate the specificity of used antibodies, we compared the protein expression levels as needed in the brain (hippocampus and cortex) and peripheral tissues (skeletal muscles and adipose tissues) as positive and negative controls, respectively.

### 2.5 Statistical analysis

All statistical analyses were performed using open‐source R software (Ihaka & Gentleman, 1996). Comparisons between diabetic and nondiabetic mice were performed using two‐tailed Welch's *t*‐tests. All graphs were produced using GraphPad Prism 9 (GraphPad Software). The statistical significance level was set at *p* < 0.05. Data are presented as dot plots and as mean ± standard error of the mean (SEM).

## 3 RESULTS

### 3.1 Some NOD mice exhibit hyperglycemia

**FIGURE 1:**
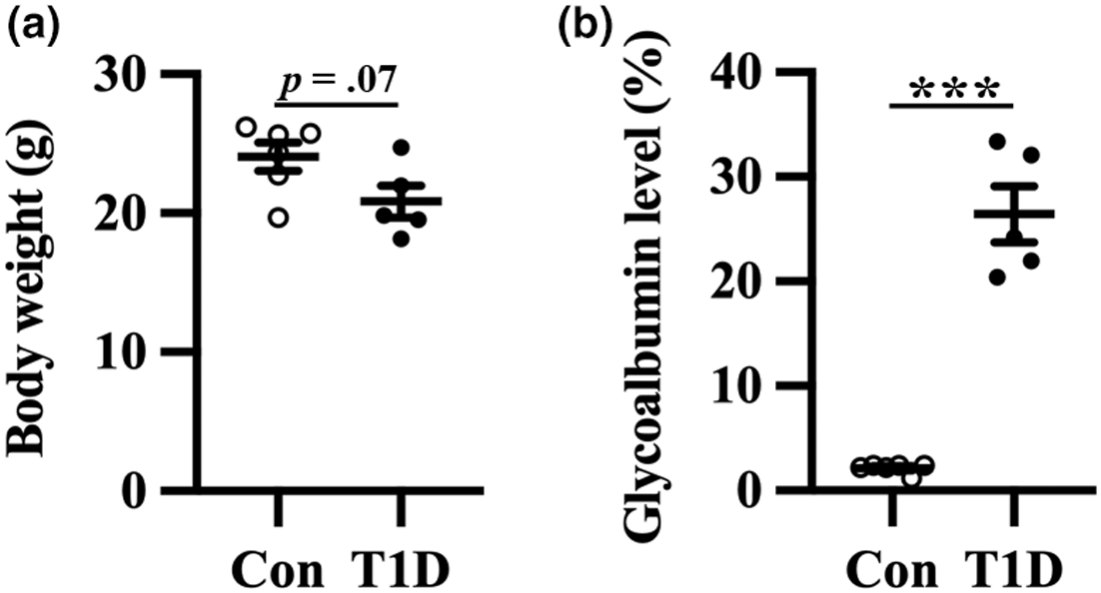
Body weight and glycoalbumin level of T1D mice and nondiabetic control mice (Con). (a) Body weight. (b) Plasma glycoalbumin level. Values are mean ± SEM; dot plot represents individual data points. *n* = 6 and *n* = 5 for Con and T1D mice, respectively. ***p < 0.001 between Con and T1D mice, Welch's *t*‐tests.

T1D mice showed a marginal decrease in body weight compared to nondiabetic mice (Figure 1a). We measured plasma glycoalbumin levels to evaluate the glycemic state. T1D mice had significantly higher plasma glycoalbumin levels than nondiabetic mice (Figure 1b). Hence, T1D mice exhibited hyperglycemia without obesity, despite daily insulin treatment, which is consistent with a previous report (Papon et al., 2012).

### 3.2 Expression of synaptic markers in the hippocampus and cortex of T1D and nondiabetic mice

**FIGURE 2:**
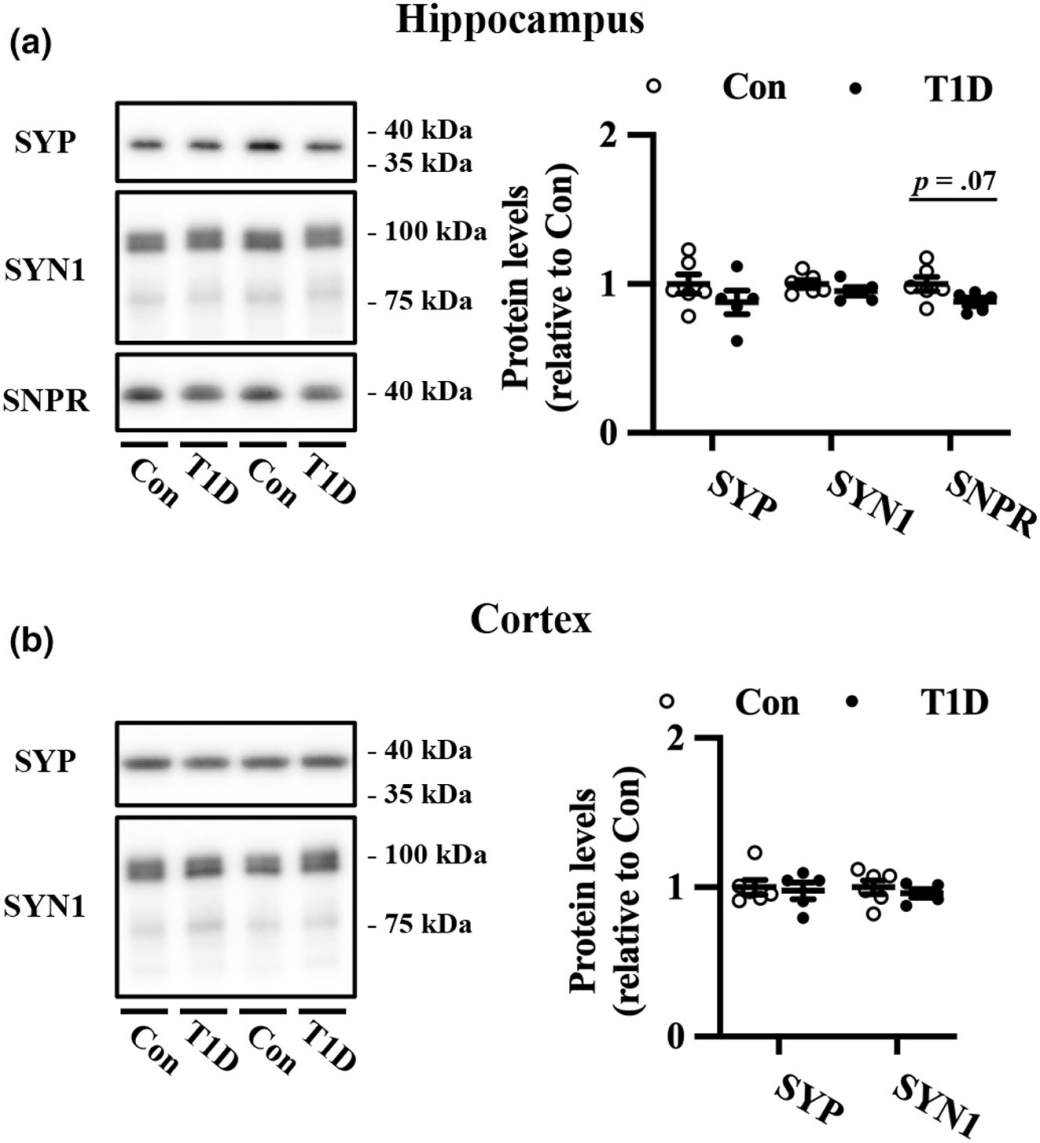
Expression levels of synaptic proteins in the hippocampus and cortex of T1D mice and nondiabetic control mice (Con). Representative immunoblots and quantification of SYN1, SNPR, and SYP protein expression levels in the hippocampus (a) and cortex (b). The protein levels were normalized to Coomassie brilliant blue staining (Figure S1). Values are mean ± SEM; dot plot represents individual data points. *n* = 6 and *n* = 5 for Con and T1D mice, respectively. Welch's *t*‐tests.

Next, we measured the protein expression of synaptic markers in the hippocampus and the cortex. T1D mice did not exhibit significant changes in SYP and SYN1 expression in the hippocampus (Figure 2a) or cortex (Figure 2b) compared with nondiabetic mice. In contrast, the expression of SNPR, a marker of mossy fiber terminals, was marginally downregulated in the hippocampus of T1D mice compared to the controls (Figure 2a).

### 3.3 T1D mice have reduced levels of excitatory synapse proteins

**FIGURE 3:**
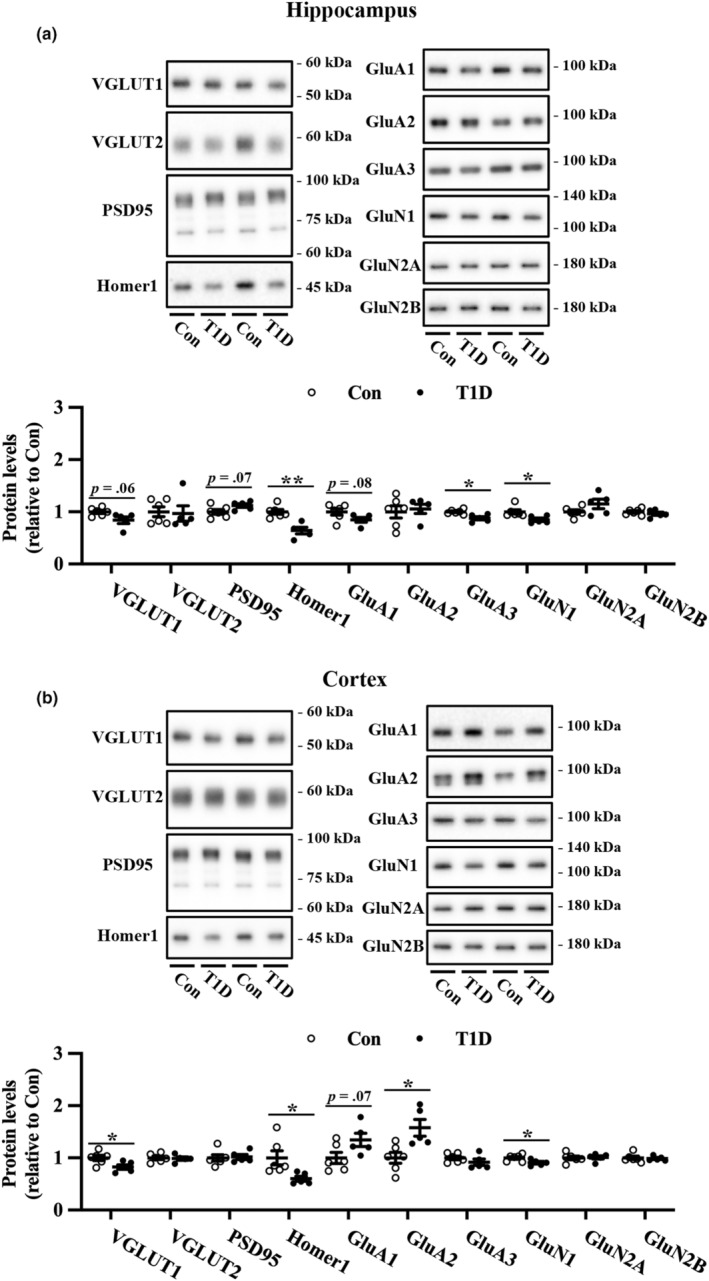
Expression levels of excitatory synapse proteins in the hippocampus and cortex of T1D mice and nondiabetic control mice (Con). Representative immunoblots and quantification of the protein expression levels of VGLUT1, VGLUT2, PSD95, Homer1, GluA1, GluA2, GluA3, GluN1, GluN2A, and GluN2B in the hippocampus (a) and cortex (b). The protein levels were normalized to Coomassie brilliant blue staining (Figure S1). Values are mean ± SEM; dot plot represents individual data points. *n* = 6 and *n* = 5 for Con and T1D mice, respectively. *p < 0.05, **p < 0.01 between Con and T1D mice, Welch's *t*‐tests.

We examined the expression of excitatory synapse proteins in the hippocampus and cortex of T1D and nondiabetic mice (Figure 3a,b). Marginally reduced VGLUT1 expression in the hippocampus and significantly decreased in VGLUT1 expression in the cortex were observed in T1D mice compared to nondiabetic mice. Conversely, we did not detect a change in VGLUT2 expression in either brain region. Although expression of the α‐amino‐3‐hydroxy‐5‐methyl‐4‐isoxazolepropionic acid receptor subunits, GluA1 and GluA2, was not significantly altered in the hippocampus, T1D mice exhibited increased GluA2 expression and a slight but nonsignificant increase in GluA1 expression in the cortex compared to nondiabetic mice. Among the NMDA receptor subunits, T1D mice exhibited significantly decreased GluN1 expression in the hippocampus and cortex compared to nondiabetic mice. In contrast, GluN2A and GluN2B expression did not change significantly in either region. Of the scaffold proteins, Homer1 expression was significantly decreased in the hippocampus and cortex of T1D mice compared to the controls. Furthermore, compared to nondiabetic mice, T1D mice exhibited a slight but nonsignificant increase in PSD‐95 expression in the hippocampus but not in the cortex.

### 3.4 T1D mice exhibit decreased expression of some inhibitory synapse proteins

**FIGURE 4:**
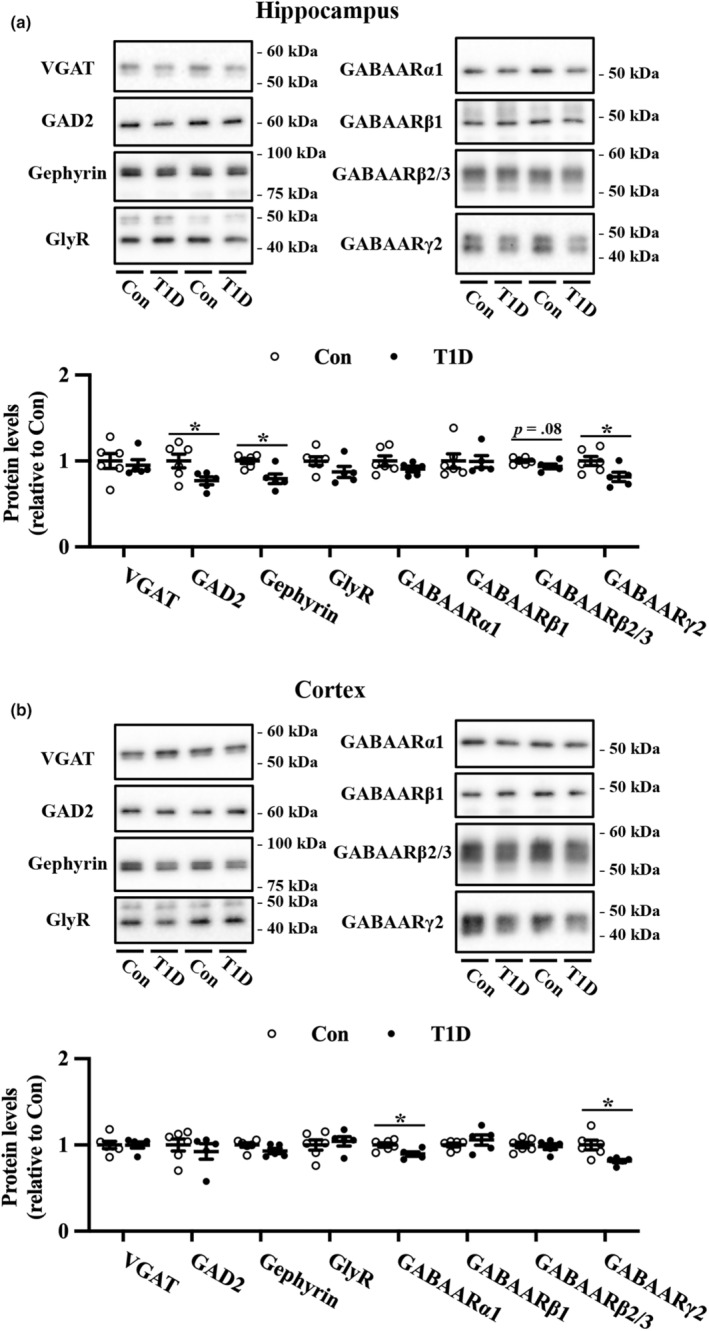
Expression levels of inhibitory synapse proteins in the hippocampus and cortex of T1D mice and nondiabetic control mice (Con). Representative immunoblots and quantification of the protein expression levels of VGAT, GAD2, Gephyrin, GlyR, GABAARα1, GABAARβ1, GABAARβ2/3, GABAARγ2 in the hippocampus (a) and cortex (b). The protein levels were normalized to Coomassie brilliant blue staining (Figure S1). Values are mean ± SEM; dot plot represents individual data points. *n* = 6 and *n* = 5 for Con and T1D mice, respectively. *p < 0.05 between Con and T1D mice, Welch's *t*‐tests.

Next, we assessed inhibitory synapse markers (Figure 4a,b). The expressions of VGAT and GAD2, both of which are predominantly localized in inhibitory presynapses, were not significantly altered in the cortex of T1D mice. In contrast, T1D mice had significantly reduced hippocampal expression of GAD2, but not VGAT, compared to nondiabetic mice. Among the GABA A receptor subunits, GABAARβ2/3 showed a marginal decrease in expression, and GABAARγ2 in the hippocampus and GABAARα1 and GABAARγ2 in the cortex were significantly downregulated in T1D mice compared to nondiabetic mice. Compared to the controls, T1D mice exhibited a decrease in the expression of gephyrin, a postsynaptic scaffold protein, in the hippocampus but not in the cortex. Conversely, the protein expression levels of glycine receptors were unchanged in the hippocampus and cortex of T1D mice compared to nondiabetic mice.

### 3.5 T1D mice have reduced expression of SAMs

**FIGURE 5:**
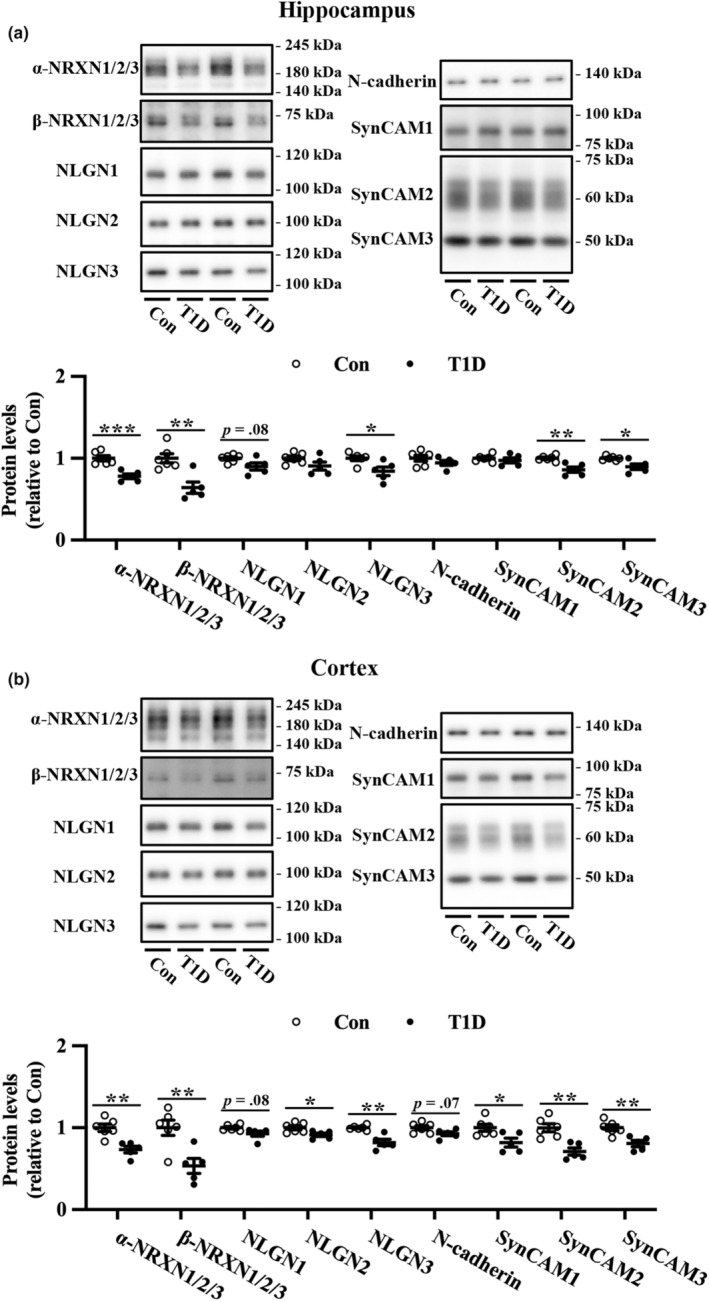
Protein expression levels of synaptic adhesion molecules in the hippocampus and cortex of T1D mice and nondiabetic control mice (Con). Representative immunoblots and quantification of the protein expression levels of NRXN1/2/3, NLGN1/2/3, N‐cadherin, and SynCAM1/2/3 in the hippocampus (a) and cortex (b). The protein levels were normalized to Coomassie brilliant blue staining (Figure S1). Values are mean ± SEM; dot plot represents individual data points. *n* = 6 and *n* = 5 for Con and T1D mice, respectively. *p < 0.05, **p < 0.01, ***p < 0.001 between Con and T1D mice, Welch's *t*‐tests.

To investigate whether T1D affects the expression of SAMs, we measured the levels of these proteins in the hippocampus and cortex of T1D and nondiabetic mice (Figure 5a,b). In T1D mice, the expression of α‐ and β‐NRXN1/2/3, which are pre‐SAMs, was significantly reduced in the hippocampus and cortex compared to the controls. T1D mice exhibited a significant decrease in NLGN3 expression and a slight but not significant decrease in NLGN1 expression in the hippocampus compared to nondiabetic controls. In the cortex, T1D mice exhibited a significant reduction in NLGN2 and NLGN3 expression, and a slight but not significant decrease in NLGN1 expression. Furthermore, compared to controls, SynCAM2 and SynCAM3 expression was downregulated in the hippocampus of T1D mice. In addition, T1D mice exhibited a significant decrease in SynCAM expression and a slight but not significant decrease in N‐cadherin expression in the cortex compared to nondiabetic mice.

## 4 DISCUSSION

In this study, we found that T1D mice had reduced expression of some synaptic proteins and SAMs in the hippocampus and cortex. This is the first study to investigate the effect of T1D on the expression of synaptic proteins and SAMs in these brain regions in detail.

In our study, T1D mice did not exhibit significant alterations in SYP and GluN2B protein expression in the hippocampus or cortex compared to nondiabetic mice. In contrast, a previous study reported that hippocampal SYP and GluN2B protein levels were reduced in streptozotocin‐treated rats (Duarte et al., 2009; Gardoni et al., 2002; Hou et al., 2012; Luca et al., 1999). This inconsistency may be a result of differences in rodent models (NOD mice vs. streptozotocin‐treated rats) or the time after T1D onset was studied. Alternatively, previous studies (Duarte et al., 2009; Gardoni et al., 2002; Luca et al., 1999), except for one (Hou et al., 2012), measured SYP and GluN2B protein levels specifically in the membrane or PSD fraction; thus, the localization, but not total expression, of SYP may change in the hippocampus of T1D mice.

Our results revealed a decrease in some excitatory synapse proteins, such as VGLUT1, Homer1, and GluN1, in the hippocampus and cortex of T1D mice (Figure 3). Mice with reduced VGLUT1 expression demonstrated enhanced anxiety‐ and depression‐like behaviors and impaired memory (King et al., 2014; Tordera et al., 2007). Previous work involving the knockout of Homer1 and GluN1 has elucidated their roles in learning and psychiatric disorders (McHugh et al., 2007; Nakazawa et al., 2002; Szumlinski et al., 2005), leading us to speculate that their downregulation may contribute to T1D‐related disturbances in synaptic plasticity and memory.

The effect of T1D on inhibitory synaptic proteins remains unclear. Here, we detected decreased expression of GAD2, gephyrin, and GABAARγ2 in the hippocampus and of GABAARα1 and γ2 in the cortex. Evidence indicates that gephyrin is involved in the formation, maintenance, and plasticity of GABAergic synapses (reviewed in Tyagarajan & Fritschy, 2014). Studies using mice heterozygous for the γ2 subunit of GABAAR (Crestani et al., 1999; Earnheart et al., 2007; Shen et al., 2010) support the hypothesis that GABAergic deficits induce anxiety‐ and depression‐like behaviors, as well as cognitive impairments (reviewed in Luscher et al., 2011). Therefore, reduced gephyrin and GABAAR subunits in T1D mice may induce GABAergic deficits, followed by psychiatric disturbance and impaired memory function.

We observed a decline in NRXN, NLGN, and SynCAM expression in the hippocampus and cortex of the T1D mice. NLGNs are post‐SAMs that bind to presynapse‐localized NRXNs (reviewed in Südhof, 2017). NLGN1 is localized at glutamatergic synapses (Song et al., 1999), NLGN2 is preferentially enriched in GABAergic synapses (Varoqueaux et al., 2004), and NLGN3 is present in glutamatergic and GABAergic synapses (Budreck & Scheiffele, 2007). Knockout and mutation of NLGNs and NRXNs in mice have helped elucidate their roles in memory function and psychiatric status (Anderson et al., 2015; Blundell et al., 2009, 2010; Dachtler et al., 2014; Etherton et al., 2009; Grayton et al., 2013; Liang et al., 2015; Radyushkin et al., 2009; Tabuchi et al., 2007). Furthermore, SynCAMs are localized at presynapses and postsynapses and play a crucial role in synaptic plasticity and memory function (Biederer et al., 2002; Fogel et al., 2007; Park et al., 2016; Robbins et al., 2010). Therefore, decreased expression of these SAMs may be involved in T1D‐related brain dysfunction.

Although numerous SAMs have been identified, we selected NRXNs, NLGNs, N‐cadherin, and SynCAMs as representative SAMs in this study because (1) substantial evidence suggests that knockout of these genes elicits synapse dysfunction, neuropsychiatric abnormalities, and memory impairment (Anderson et al., 2015; Blundell et al., 2010; Bozdagi et al., 2010; Dachtler et al., 2014; Etherton et al., 2009; Grayton et al., 2013; Liang et al., 2015; Nikitczuk et al., 2014; Park et al., 2016; Robbins et al., 2010), all of which partly resemble the phenotypes of T1D rodent models (Erion et al., 2014; Ogrodnik et al., 2019; Stranahan, Arumugam, et al., 2008, Stranahan, Norman, et al., 2008); (2) knockout‐ or blocking peptide‐validated antibodies are commercially available; (3) clear immunoblots were obtained for quantitative analysis. Several studies have shown that various other SAMs are associated with synaptic and brain functions (reviewed in Südhof, 2018). Numerous splice variants of SAMs have also been identified; therefore, transcriptome and proteome analyses may be suitable for further studies. SAMs have been underscored as pharmacological targets for treating neuropsychiatric and neurological disorders (Suzuki et al., 2020; van der Kooij et al., 2014). Hence, additional studies focusing on SAMs may reveal their potential as therapeutic targets for T1D‐related brain dysfunctions.

The current study has some limitations in addition to those previously mentioned. First, we did not measure the target protein levels in detailed brain regions of the hippocampus and cortex. Second, we did not assess the localization or protein–protein interactions of synaptic proteins and SAMs. Finally, the causal relationship between brain function, synaptic proteins, and SAMs in T1D remains unclear, suggesting that further studies using loss‐ and gain‐of‐function approaches are required.

In conclusion, our study demonstrated a partial decrease in synaptic proteins and SAMs in the hippocampus and cortex of a T1D mouse model, providing novel molecular‐level insights into synaptic dysregulation in T1D.

## AUTHOR CONTRIBUTIONS

*Conceptualization*: Takumi Yokokawa and Kohei Kido. *Formal analysis*: Takumi Yokokawa and Kohei Kido. *Investigation*: Takumi Yokokawa and Kohei Kido. *Writing—original draft preparation*: Takumi Yokokawa. *Writing—review and editing*: Kohei Kido, Tatsuya Hayashi, Koji Sato, and Satoshi Fujita. *Supervision*: Tatsuya Hayashi, Koji Sato, and Satoshi Fujita. *Funding acquisition*: Takumi Yokokawa, Tatsuya Hayashi, Koji Sato, and Satoshi Fujita. All authors have read and agreed to the published version of the manuscript.

## FUNDING INFORMATION

This work was supported by the Japan Society for the Promotion of Science Grants in Aid for Scientific Research (Nos. 16J10577, 19K24330, and 20K19498 to Takumi Yokokawa and 17H02183 to Satoshi Fujita) and Takeda Research Support (TKDS20170531015 to Tatsuya Hayashi).

## CONFLICT OF INTEREST STATEMENT

The authors declare no conflicts of interest, financial or otherwise.

## ETHICS STATEMENT

All animal care procedures were approved by the Committee on Animal Care at Ritsumeikan University.

## Supporting information


Figure S1.
