# Supplementary figures and images for: Altered expression of synaptic proteins and adhesion molecules in the hippocampus and cortex following the onset of diabetes in nonobese diabetic mice
Source: Physiol Rep. 2023 Apr 20;11(8):e15673. doi: 10.14814/phy2.15673 (PMC10116544; doi:10.14814/phy2.15673)

**A****Hippocampus****CBB**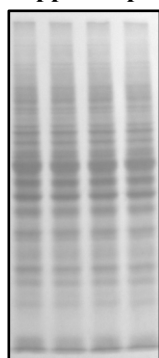

Con T1D Con T1D

**B****Cortex****CBB**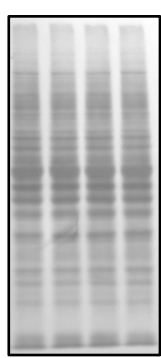

Con T1D Con T1D

Supplement: Supplementary file 1 — Figure S1. [file PHY2-11-e15673-s001.pdf]
